# Supplementary material for: Arousal dependent modulation of thalamo-cortical functional interaction
Source: Nat Commun. 2018 Jun 25;9:2455. doi: 10.1038/s41467-018-04785-6 (PMC6018110; doi:10.1038/s41467-018-04785-6)
Supplement: Supplementary file 1 — Supplementary Information [file 41467_2018_4785_MOESM1_ESM.pdf]

Arousal dependent modulation of thalamo-cortical functional interaction

Stitt et al.

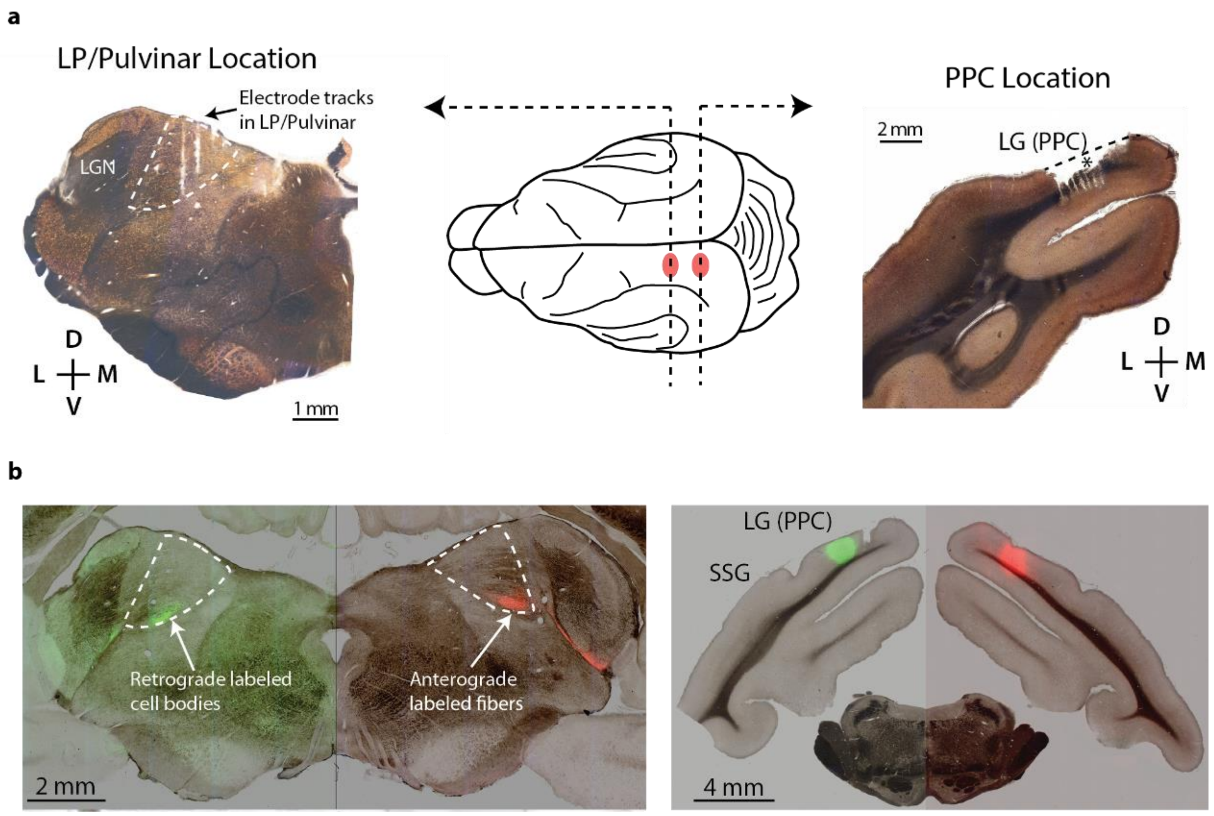

Supplementary Figure 1 / Histological confirmation of multielectrode array recording sites in anatomically connected sites of PPC and LP/Pulvinar.

a, Coronal sections of LP/Pulvinar (left) and PPC (right) stained for cytochrome oxidase were used for verifying the location of multielectrode array recording sites. The middle panel shows a schematic representation of the ferret brain, with the approximate location of LP/Pulvinar and PPC electrode implantation shown in red. Left: A representative section showing electrode tracks and recording site in the LP/Pulvinar. Right: A representative section showing the recording site of a PPC microelectrode array. The location of the electrode implantation is illustrated by the damage induced from removing the microelectrode array (indicated by *). **b**, The location of LP/Pulvinar and PPC microelectrode implantation overlapped with patterns of anatomical connectivity outlined in previous tracing experiments, indicating that electrophysiological data were obtained from reciprocally connected regions of thalamus and cortex. Abbreviations: LG (lateral gyrus), SSG (suprasylvian gyrus).

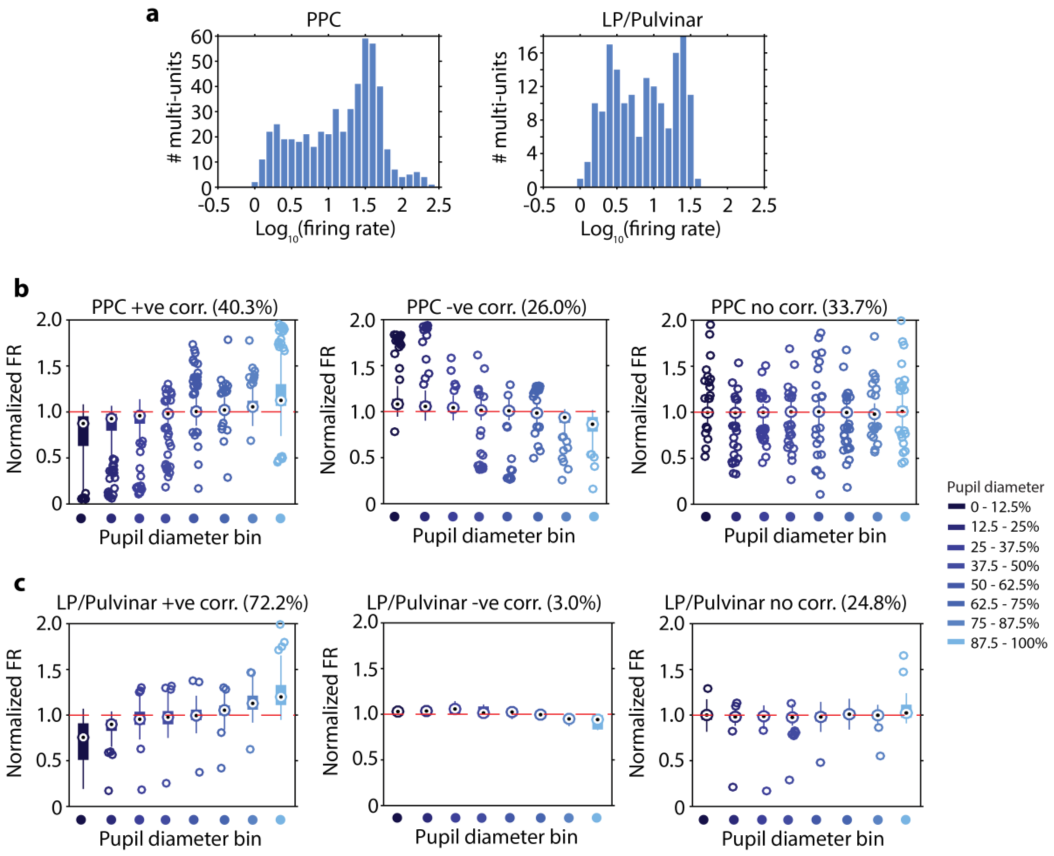

Supplementary Figure 2 / Pupil-linked arousal dependent changes in PPC and LP/Pulvinar firing rate. a, Distribution of multi-unit firing rates in PPC (n = 519) and LP/Pulvinar (n = 169). **b**, Population box plots of PPC firing rate across each pupil diameter bin. PPC multi-units were sorted into three populations; units that significantly increased firing rate with increasing pupil diameter (40.3% of units), units that significantly decreased firing rate with increasing pupil diameter (26.0% of units), and units where there was no relationship between firing rate and pupil diameter (33.7% of units). **c**, The same analysis as **b** performed for LP/Pulvinar multi-unit spiking activity. In contrast to PPC, where units displayed both increases and decreases in firing rate with pupil-linked arousal, the vast majority of multi units in LP/Pulvinar (72.2%) displayed significantly increased firing rate with increasing pupil diameter. In addition, very few (3.0%) units displayed a decrease in firing rate with pupil-linked arousal.

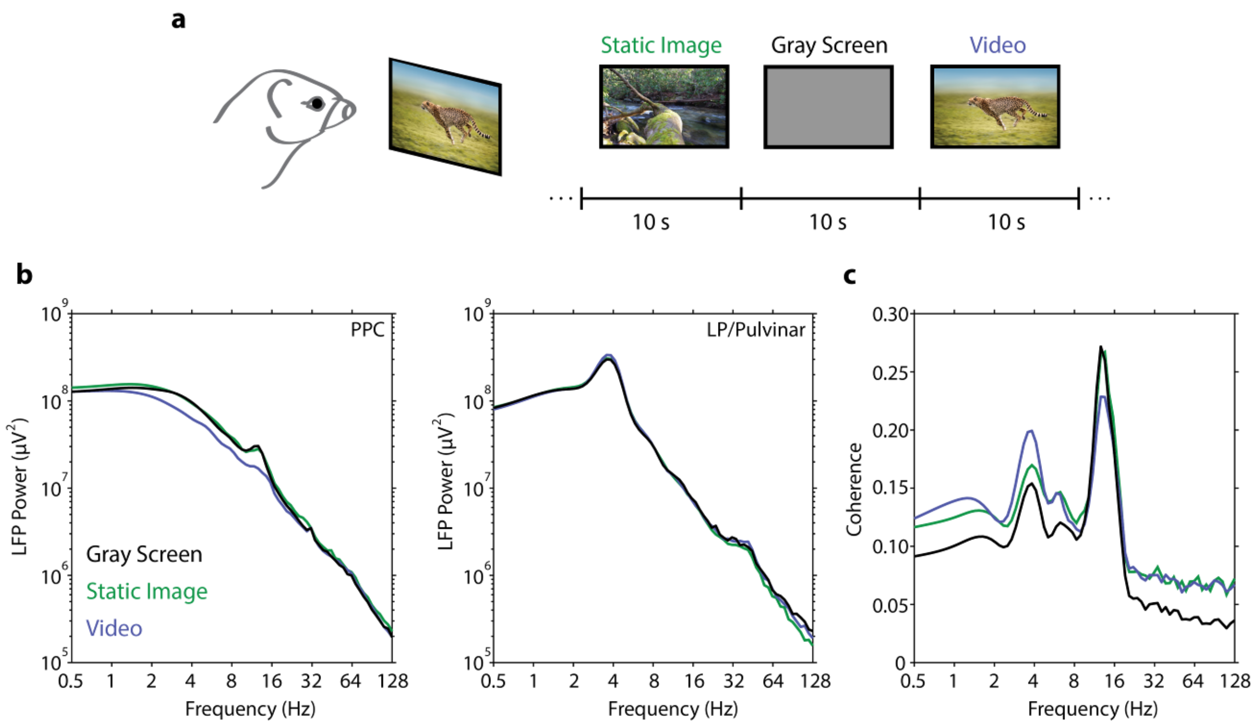

Supplementary Figure 3 / PPC and LP/Pulvinar LFP power spectra under different visual stimulus conditions. a, Animals were presented with a library of static images and videos that were randomly interleaved. During the inter-stimulus interval a gray screen was presented. The image "Running Cheetah" by Freder is licensed under the Standard iStock Photo License (Getty Images). **b**, The mean LFP power spectrum in PPC and LP/Pulvinar for the different stimulus conditions. Note the presence of a prominent peak in the power spectrum at around 12-17 Hz in PPC, which was strongest in the gray screen and static images conditions. LP/Pulvinar LFP spectra were marked by a large peak in the theta frequency band (3.3-4.5 Hz). **c,** The coherence spectrum between co-recorded signals in PPC and LP/Pulvinar. Note the peak in coherence in the 12-17 Hz frequency band that reduces in magnitude for the video condition. Given that LFP signal power and thalamo-cortical coherence in this frequency range was reduced following visual stimulation, we interpreted the frequency band centered around 14Hz to reflect the endogenous alpha frequency in the ferret. This is in line with a mechanistic definition of the alpha rhythm as a thalamo-cortically generated oscillation that reduces in amplitude when networks are engaged in processing sensory information.

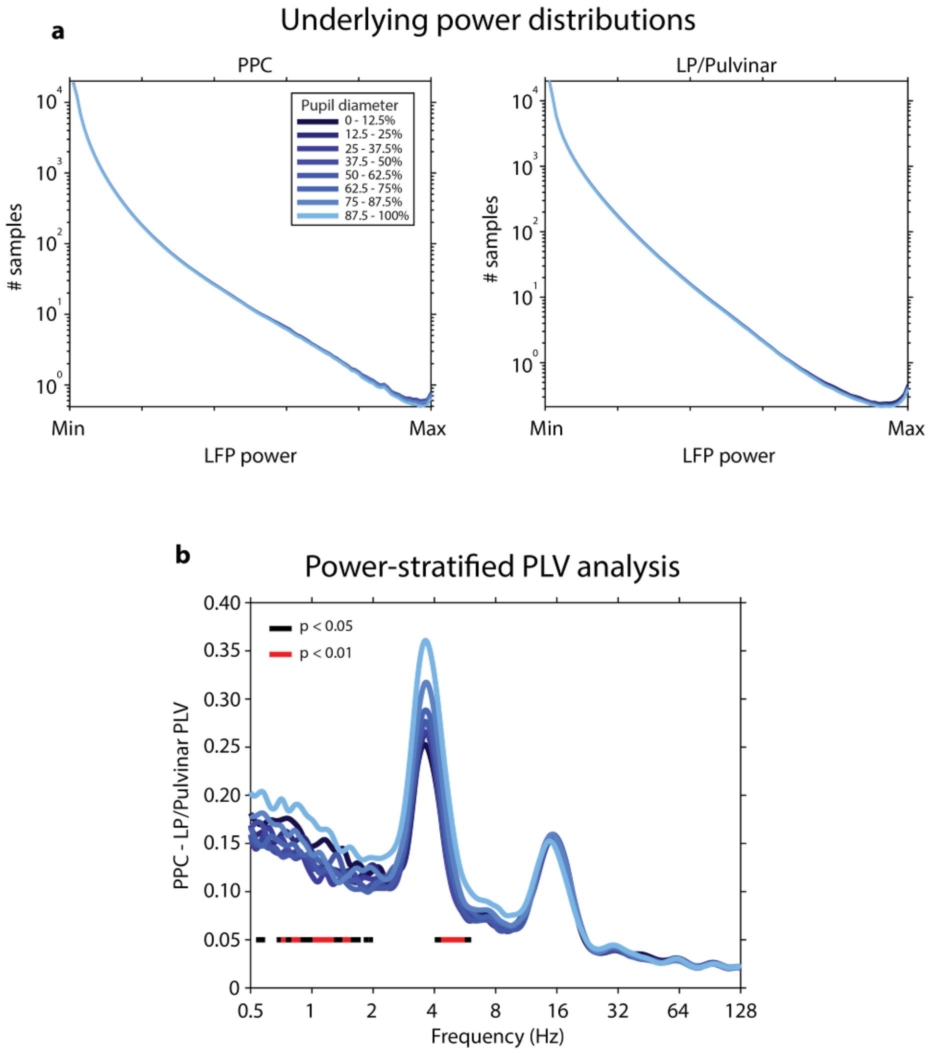

Supplementary Figure 4 / Thalamo-cortical phase synchronization with matched power distributions. a, LFP data were subsampled within each frequency band to match the power distributions across all pupil diameter bins. **b**, Thalamo-cortical phase synchronization based on LFP data that were matched for power across pupil diameter bins. The color bars below PLV plots illustrate frequency bands that display a significant effect of pupil diameter (one-way ANOVA, FDR corrected P values). PLV in the theta frequency band remains significant after subsampling data to match power values across conditions. Power matched PLV analysis in the alpha band shows no significant change across pupil diameter bins. However, this result is in contrast to thalamo-cortical spike-spike correlation analysis, which displays a clear effect in the alpha band (Supplementary Figure 5). Given that spike correlations more closely reflect the physiological processes underlying thalamo-cortical functional interaction, we argue that the lack

of significant modulation of PLV across pupil diameter bins in the alpha band is an artifact of the power matching procedure.

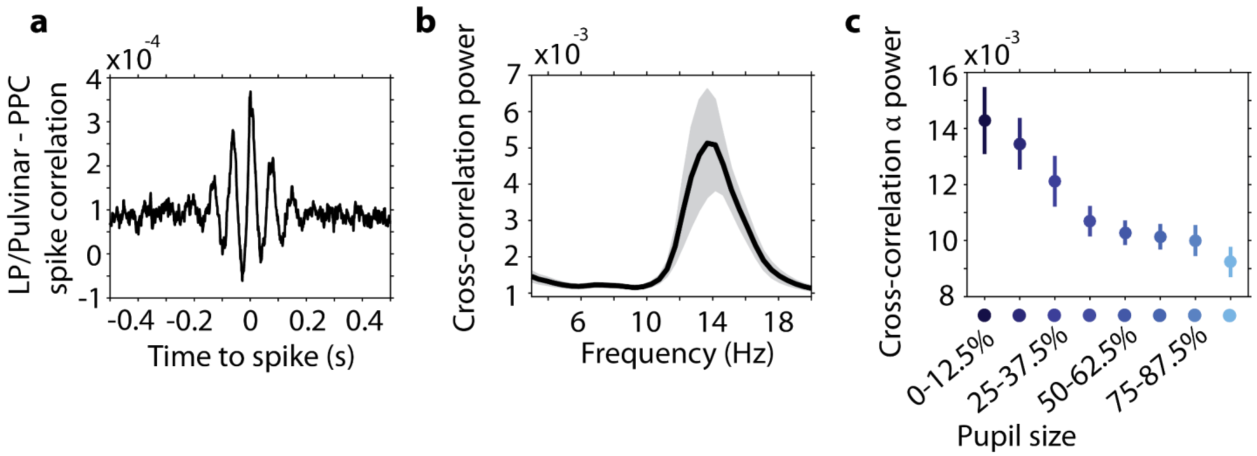

Supplementary Figure 5 / Thalamo-cortical spike correlation as a function of pupil diameter. a, Population average spike cross-correlation measured between LP/Pulvinar and PPC recording sites. Note the synchronous oscillatory structure occurring within the alpha frequency band (period of 68 ms ~14.7 Hz). **b,** The mean power spectrum (± SEM) of thalamo-cortical spike cross-correlation displays a prominent peak in the alpha band. **c,** Alpha oscillatory power in thalamo-cortical spike cross correlation significantly decreased with pupil dilation ($P = 4.1^{-6}$ one-way ANOVA, $r = -0.42$).

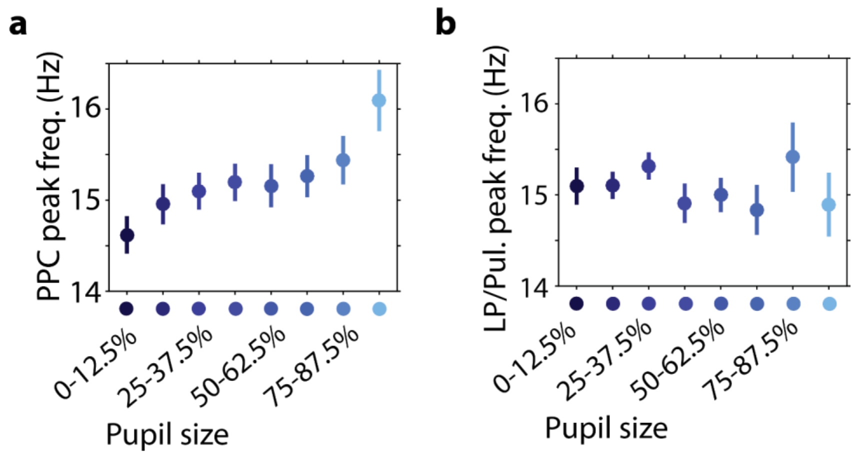

Supplementary Figure 6 / Endogenous alpha frequency speeds up in cortex with arousal. a-b, Alpha frequency was measured by taking the peak of spike-LFP phase synchronization spectra within PPC (**a**), and LP/Pulvinar (**b**). Note that peak alpha frequency speeds up in PPC with increasing pupil diameter (P = 0.003 one-way ANOVA). In contrast, peak alpha frequency in LP/Pulvinar displays no relationship to changes in pupil size (P = 0.85 one-way ANOVA).

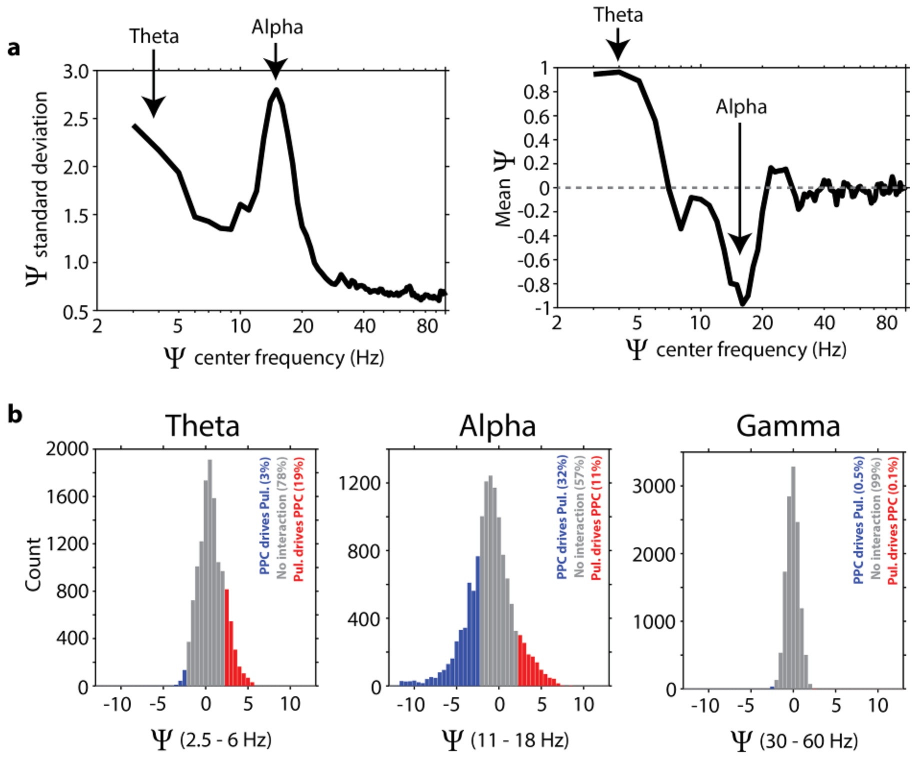

Supplementary Figure 7 / Phase slope index (Ψ) uncovers reciprocal thalamo-cortical effective connectivity in the theta and alpha frequency bands. a, The mean and standard deviation of population level phase slope index (PSI) analyses. PSI was initially computed on LP/Pulvinar and PPC LFP signals in a 4 Hz sliding window in the frequency domain (1-100 Hz in 1 Hz steps). We observed both significant drivers and receivers in each recording, with values greater than 2 indicating that LP/Pulvinar significantly drives PPC, and values lower than -2 indicating that PPC significantly drives LP/Pulvinar ($p < 0.05$). The standard deviation of PSI values was therefore used to infer the width of the PSI distribution, or the extent to which each carrier frequency displays significant drivers or receivers. At the same time the mean indicates if thalamo-cortical (positive PSI values) or cortico-thalamic (negative PSI values) effective connectivity dominates population level PSI analyses. We observed the largest PSI standard deviation in the theta and alpha frequency bands, indicating these are the carrier frequency bands of thalamo-cortical effective connectivity. The mean of the PSI distribution shifted from positive in the theta band to negative in the alpha band, with all other frequencies converging on zero. Together, these results indicate there is generally greater effective connectivity in the theta and alpha bands, but that these frequency bands reflect effective connectivity in opposing directions, with theta band effective connectivity indicating LP/Pulvinar drives PPC, and alpha band

effective connectivity indicating PPC drives LP/Pulvinar. **b,** Population level PSI analysis computed on all channel pairs for the theta (2.5-6 Hz), alpha (11-18 Hz), and gamma (30-60 Hz) frequency bands. In each histogram, red bars indicate the proportion of channel pairs where LP/Pulvinar significantly drives PPC, gray bars indicate no significant interaction, and blue bars indicate where PPC significantly drives LP/Pulvinar. The percentage of total channel pairs for each sub-group are given as insets in each figure. Note that theta and alpha PSI values show wider distributions, and that the center of mass is shifted away from zero. In contrast, gamma PSI values are closely centered around zero, indicating this frequency range does not mediate thalamo-cortical effective connectivity.

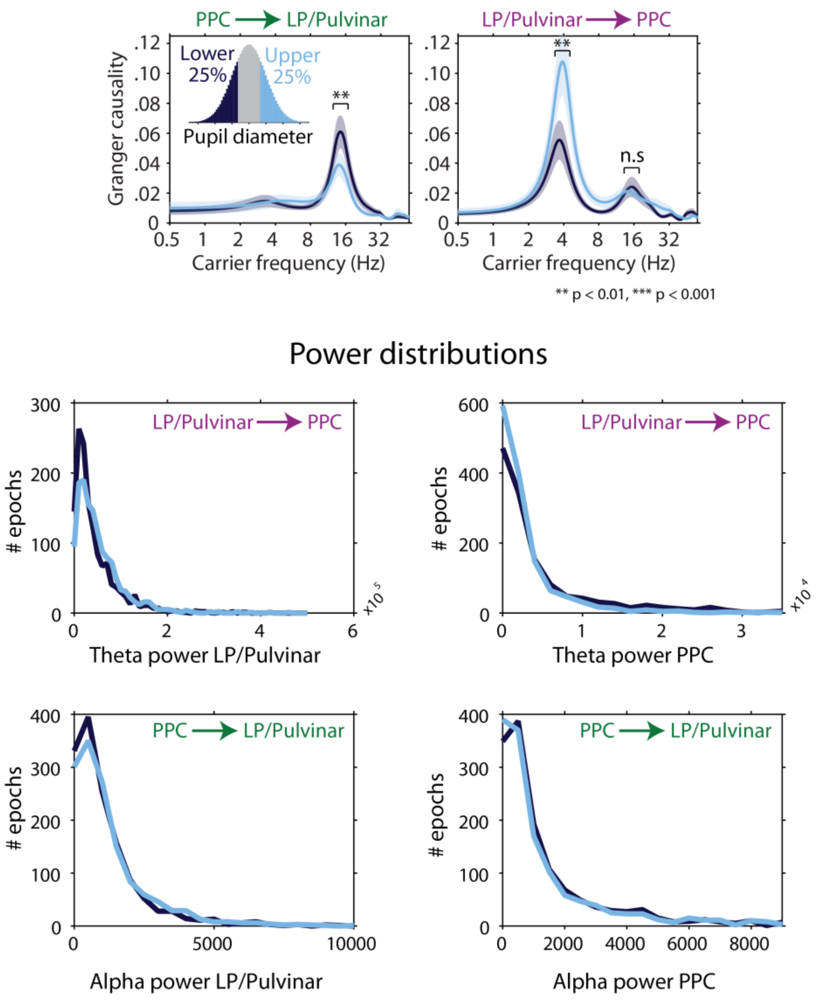

Supplementary Figure 8 / Power matched Granger causality analysis. Granger causality was measured for time periods where the pupil diameter was small (< 25%, dark blue), and large (> 25%, light blue), respectively. Data were subsampled to match power distributions for the theta and alpha frequency bands between conditions. Given that the theta band was the main carrier frequency of thalamus-to-cortex effective connectivity, we matched power distributions in the theta band while computing LP/Pulvinar to PPC Granger causality. Similarly, since alpha was the predominant carrier frequency of cortex-to-thalamus effective connectivity, we matched power distributions in the alpha band while computing PPCto LP/Pulvinar Granger causality. The power distributions of data that were used for Granger causality estimation are shown in the lower four plots.

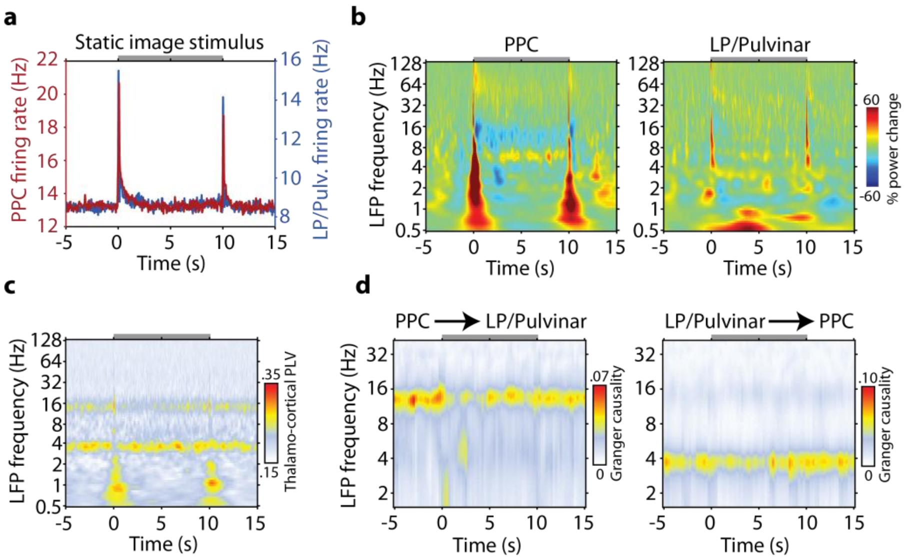

Supplementary Figure 9 / PPC and LP/Pulvinar responses to naturalistic images. a, Across session average firing rate during presentation of naturalistic images. **b**, Across session average LFP spectrogram for PPC (left) and LP/Pulvinar (right) in response to naturalistic images. LFP power was normalized by the prestimulus power (-5 to -1 seconds). **c**, Average thalamo-cortical PLV during the time course of naturalistic image presentation. **d**, Average time and spectral resolved Granger causality analysis for naturalistic image presentation. The gray bar at the top of each plot indicates the duration of naturalistic image presentation.

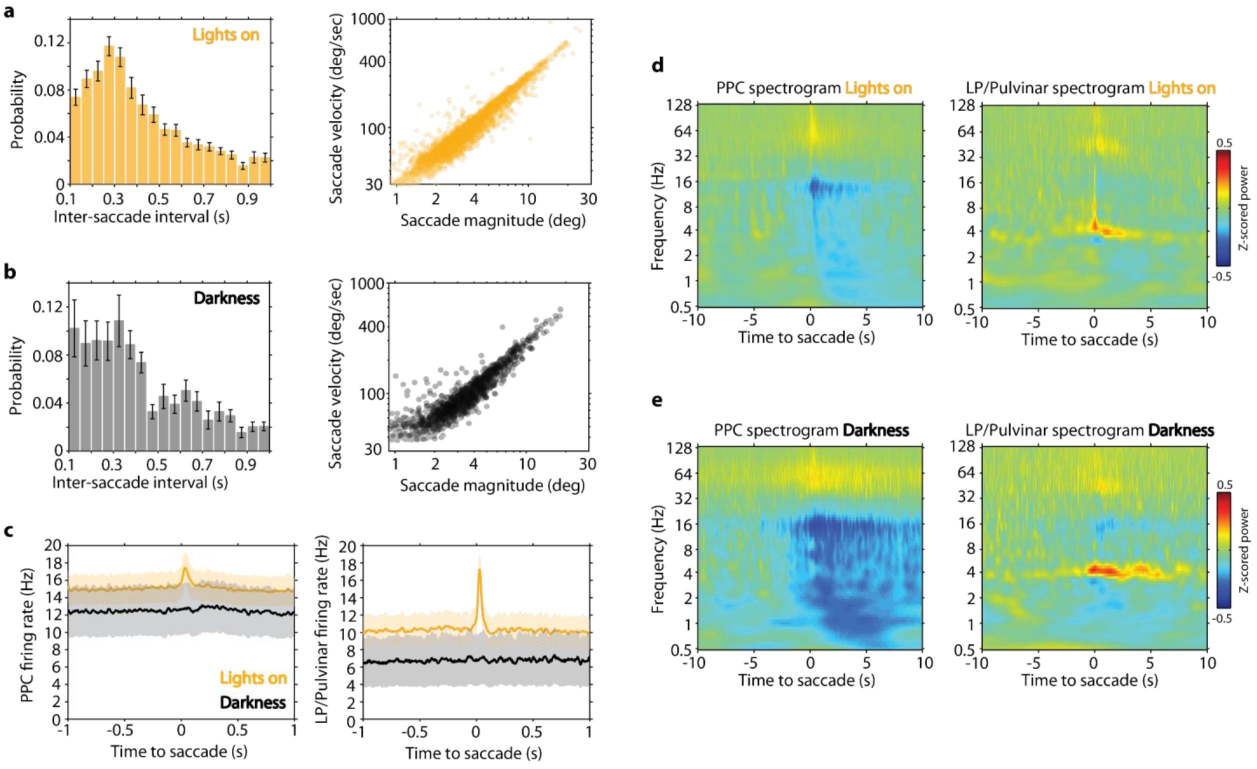

Supplementary Figure 10 / Basic saccade properties and saccade-locked measures of spiking activity and LFP power. a-b, Inter saccade interval (± SEM) and scatter plot of saccade magnitude versus peak velocity for saccades performed with the lights on (**a**) and in a dark room (**b**). **c**, Across session average firing rate in PPC (left) and LP/Pulvinar (right) time locked to the occurrence of saccades in the light and dark (shaded regions indicate ± SEM). Saccade-locked increases in firing rate in PPC and LP/Pulvinar are only present when the lights are on, suggesting that these responses depend on visual input. **d-e**, Across session average LFP spectrograms computed around the occurrence of saccades with the lights on (**d**) and in a dark room (**e**). LFP power was z-score normalized within each frequency band across the entire recording session. In contrast to the fast modulation of spiking activity during saccades, LFP power spectra in both lights on and lights off conditions reflect slower fluctuations in PPC alpha and LP/Pulvinar theta rhythms.

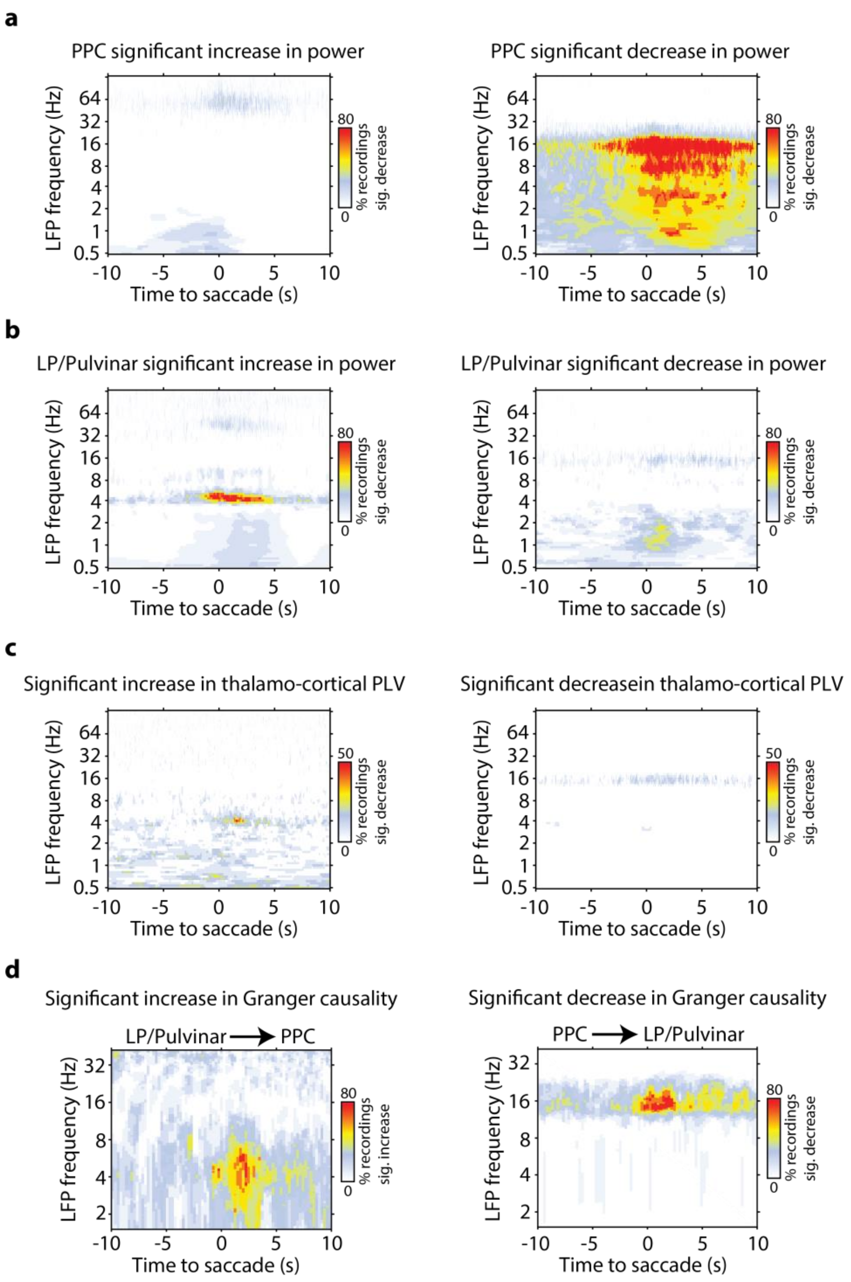

Supplementary Figure 11 / Significant modulation of thalamo-cortical power spectra and functional connectivity time locked to saccades in the dark. In each of the plots above, significance was estimated by building surrogate distributions of LFP power (**a-b**), PLV (**c**), and Granger causality (**d**) at random time points during each recording. Time and frequency points relative to the onset of saccades that deviated 2 standard deviations from the mean of randomly computed metrics were then deemed as significant deviations (P < 0.05). **a,** Most recordings in

PPC display significant reductions in alpha and low frequency LFP oscillations for the several seconds surrounding saccades. **b,** Most recordings from LP/Pulvinar display significant increases in narrowband theta oscillations coinciding with saccadic eye movements. **c,** Although thalamo-cortical PLV tended to increase in the theta band, and decrease in the alpha band around saccades, these changes were significant in only a minority of recordings. **d,** These plots show that the causal influence of cortical alpha rhythms on thalamus breaks down as animals begin to actively sample the visual environment with saccades. Conversely, thalamic causal influence on cortex in the theta band increases predominantly after animals begin to saccade.

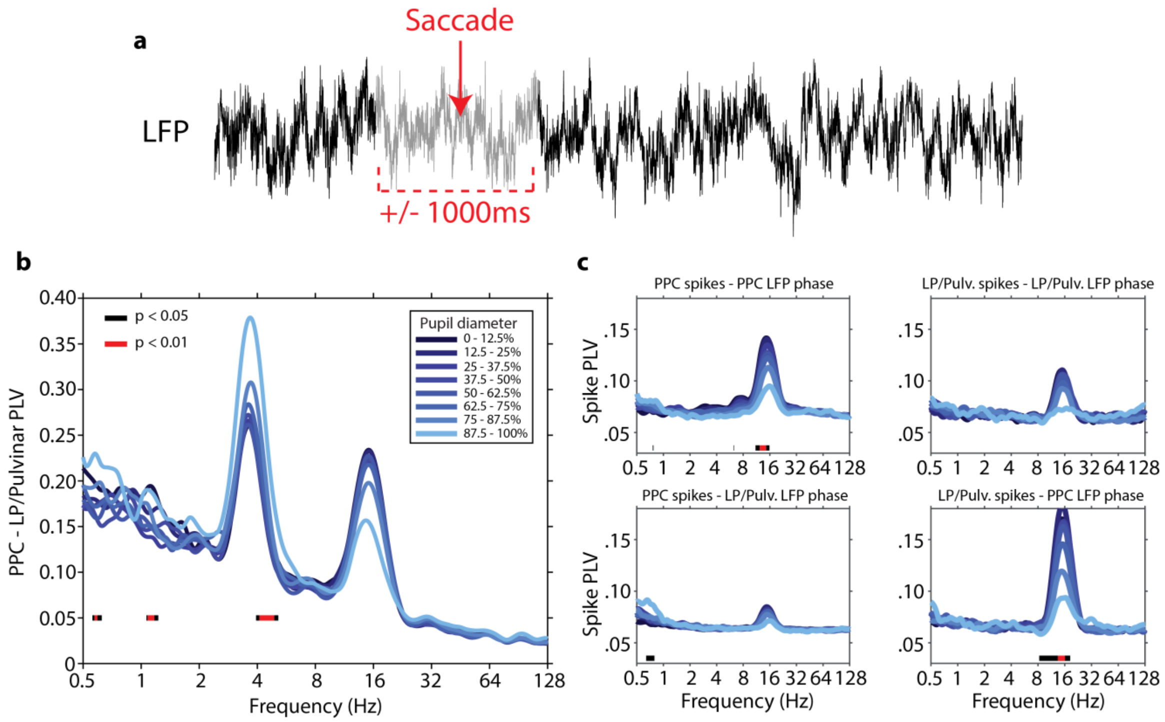

Supplementary Figure 12 / Thalamo-cortical functional connectivity analysis after controlling for peri-saccade epochs. a, To attempt to disentangle saccade- and pupil-related changes in thalamo-cortical functional connectivity, we reanalyzed LFP recordings after removing epochs 1000ms before and after saccades. **b**, PLV measured between LP/Pulvinar and PPC as a function of LFP frequency and pupil diameter after removing peri-saccadic epochs. **c**, The phase synchronization (PLV) of spiking activity to LFP rhythms recorded within the same brain structure (top row), as well as between regions (bottom-row) as a function of pupil derivative after removing peri-saccadic epochs. Note that despite removing peri-saccade epochs, the main findings of theta synchronization and alpha spike-LFP phase synchronization remain significant. Despite pupil linked arousal dependent changes in alpha thalamo-cortical synchronization not reaching significance, there was a tendency for larger pupil diameters to correlate with reduced synchronization.

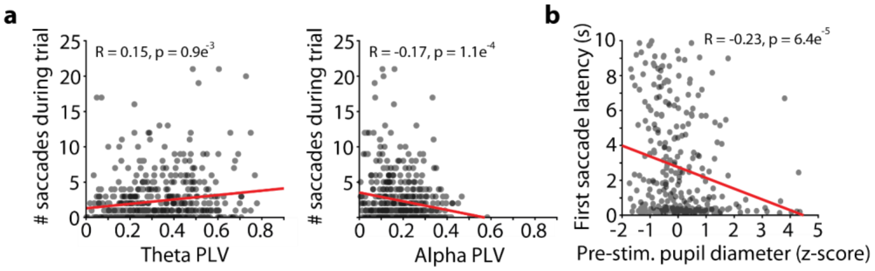

Supplementary Figure 13 / Correlation of thalamo-cortical functional connectivity and prestimulus pupil diameter to oculomotor behavior while viewing naturalistic images. a, Correlation of thalamo-cortical phase synchronization in the theta (left) and alpha (right) carrier frequency bands and the number of saccades performed during presentation of naturalistic images. Theta PLV displays a significant positive correlation with saccadic sampling of stimuli, whereas alpha PLV displays a significant negative correlation. **b**, Correlation of pre-stimulus pupil diameter to the latency of the first saccade for subsequent naturalistic image stimulus presentation. Significant negative correlation illustrates that animals sample incoming visual stimuli more rapidly when they are in a more aroused state.

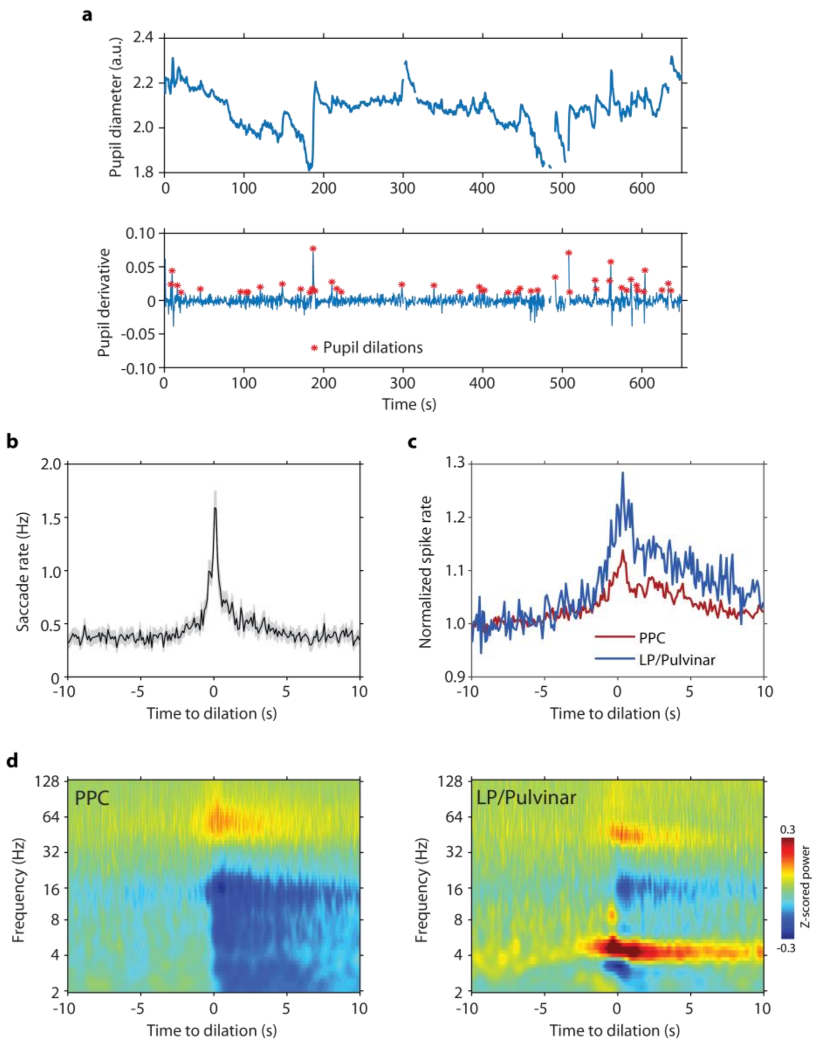

Supplementary Figure 14 / Transient pupil dilations lead to the reorganization of oculomotor behavior and neural activity in PPC and LP/Pulvinar. a, Pupil diameter (top) and pupil diameter first derivative (bottom) for one example recording. Transient pupil dilations were detected by finding local peaks in pupil diameter derivative time series. Discontinuities in plots indicate where data have been removed around the occurrence of large-amplitude saccades or eye-blinks. **b**, Mean saccade rate (± SEM) time locked to the occurrence of pupil dilations. **c**, Average multiunit spike rate in PPC and LP/Pulvinar time locked to pupil dilations. **d**, LFP power spectrograms in PPC (left) and LP/Pulvinar (right) time locked to pupil dilations. LFP power was z-score normalized across the entire recording session.
